# Supplementary material for: Photosynthetic Performance, Carbohydrate Partitioning, Growth, and Yield among Cassava Genotypes under Full Irrigation and Early Drought Treatment in a Tropical Savanna Climate
Source: Plants (Basel). 2024 Jul 25;13(15):2049. doi: 10.3390/plants13152049 (PMC11313790; doi:10.3390/plants13152049)
Supplement: Supplementary file 1 [file plants-13-02049-s001.zip › plants-3118236-supplementary.pdf]

Supplementary Tables and Figures

Table S1. The meteorological parameters including monthly mean light intensity, air temperature and relative humidity (RH), total rainfall and number of rainy days in the cassava field during August, 2021 to August, 2022.

Monthly	Light intensity (µmol photon $m^{-2} s^{-1}$)	Temperature (°C)			RH (%)	Total rainfall (mm)	Rainy day number
		max.	min	mean			
August, 2021	486.6	34.0	24.7	28.3	75.6	112.9	11.0
September, 2021	385.3	32.1	23.9	26.9	85.2	246.7	23.0
October, 2021	394.6	32.0	23.1	26.8	78.6	127.5	13.0
November, 2021	423.3	32.4	20.7	26.0	66.1	0.0	0.0
December, 2021	411.1	30.6	16.6	23.3	58.8	0.0	0.0
January, 2022	417.5	32.7	18.1	24.8	59.6	1.5	3.0
February, 2022	382.6	30.9	19.3	24.6	65.9	34.3	7.0
March, 2022	467.4	34.9	23.5	28.3	65.4	56.0	9.0
April, 2022	467.3	34.9	23.1	28.3	60.9	58.3	6.0
May, 2022	427.8	32.3	23.3	27.8	84.1	135.8	21.0
June, 2022	512.3	34.6	24.5	29.1	77.8	148.8	17.0
July, 2022	417.8	33.1	24.5	28.2	87.8	244.4	21.0
August, 2022	430.3	32.0	23.9	27.4	88.5	189.9	17.0

Table S2. Chlorophyll fluorescence parameters of cassava genotypes Rayong 9 (RY9), Rayong 72 (RY72), Kasetsart 50 (KU50), CMR38-125-77, CMR35-91-63 at the plant age of 3, 4, 5, 6, and 12 months after planting (MAP). Maximum photochemical quantum yield of PSII (Fv/Fm), effective quantum yield of PSII photochemistry (ΦPSII), and electron transport rate (ETR) were measured in cassava growing under the control (continuous irrigation from 0MAP to 12MAP) and drought (irrigation was withheld for 60 days in the dry season during 4MAP and 5MAP then rewatered until 12MAP) treatment. Different capital letters indicated significant ($p < 0.05$) differences among the age of plants, whereas those among genotypes are denoted with different lowercase letters in each plant age.

Parameter	Chlorophyll fluorescent						Mean
	RY9	RY72	KU50	CMR38-125-77	CMR35-91-63	CM523-7	
**Control plants**							
**Fv/Fm**							
3 MAP	0.88 ± 0.01 A	0.85 ± 0.01 AB	0.86 ± 0.01 AB	0.89 ± 0.02 AB	0.87 ± 0.01 AB	0.86 ± 0.01 AB	**0.87 B**
4 MAP	0.84 ± 0.01 B	0.83 ± 0.01 B	0.83 ± 0.01 B	0.84 ± 0.01 B	0.83 ± 0.01 B	0.83 ± 0.01 B	**0.83 C**
5 MAP	0.83 ± 0.01 B	0.84 ± 0.01 B	0.83 ± 0.01 B	0.84 ± 0.01 AB	0.83 ± 0.01 B	0.84 ± 0.01 B	**0.83 C**
6 MAP	0.89 ± 0.01 A	0.88 ± 0.01 A	0.87 ± 0.01 A	0.88 ± 0.01 A	0.91 ± 0.01 A	0.89 ± 0.02 A	**0.89 A**
12 MAP	0.83 ± 0.01 B	0.83± 0.01 B	0.82 ± 0.01 B	0.83 ± 0.01 B	0.83 ± 0.01 B	0.83 ± 0.01 B	**0.83 C**
**Mean**	**0.86**	**0.85**	**0.85**	**0.86**	**0.86**	**0.85**	
**F-test**	17.17	5.64	5.79	5.55	13.28	6.01	**48.18**
***P*-value**	< 0.01	< 0.01	< 0.01	< 0.01	< 0.01	< 0.01	**< 0.01**

Parameter	Chlorophyll fluorescent						Mean
	RY9	RY72	KU50	CMR38-125-77	CMR35-91-63	CM523-7	
**ΦPSII**							
3 MAP	0.55 ± 0.01 A	0.55 ± 0.05 A	0.62 ± 0.01 A	0.58 ± 0.07 A	0.59 ± 0.05 A	0.62 ± 0.05 A	**0.58 A**
4 MAP	0.56 ± 0.05 A	0.55 ± 0.05 A	0.58 ± 0.03 A	0.60 ± 0.06 A	0.64 ± 0.05 A	0.62 ± 0.04 A	**0.59 A**
5 MAP	0.37 ± 0.02 BCab	0.33 ± 0.02 Bab	0.26 ± 0.01 BCc	0.39 ± 0.03 ABa	0.34 ± 0.02 Bab	0.35 ± 0.04 Bab	**0.34 B**
6 MAP	0.40 ± 0.01 B	0.32 ± 0.02 B	0.31 ± 0.01 B	0.35 ± 0.04 B	0.36 ± 0.03 B	0.35 ± 0.01 B	**0.35 B**
12 MAP	0.28 ± 0.02 C	0.26± 0.01 B	0.22 ± 0.01 C	0.30 ± 0.03 B	0.26 ± 0.01 B	0.26 ± 0.03 B	**0.26 C**
**Mean**	**0.44**	**0.41**	**0.41**	**0.45**	**0.44**	**0.44**	
**F-test**	21.03	18.10	99.99	7.35	24.22	22.39	**117.40**
***P*-value**	< 0.01	< 0.01	< 0.01	< 0.01	< 0.01	< 0.01	**< 0.01**
**ETR**	**(μmol e- m^{-2} s^{-1})**						
3 MAP	342 ± 9 A	316 ± 19 A	350 ± 7 A	335 ± 26 A	319 ± 32 A	372 ± 33 A	**339 A**
4 MAP	331 ± 42 A	279 ± 22 A	310 ± 23 A	340 ± 43 A	342 ± 34 A	330 ± 52 A	**322 A**
5 MAP	193 ± 8 B	207 ± 10 B	156 ± 15 B	192 ± 9 B	190 ± 27 B	166 ± 11 B	**184 B**
6 MAP	197 ± 8 B	165 ± 5 BC	162 ± 13 B	166 ± 6 B	191 ± 12 B	189 ± 17 B	**178 BC**
12 MAP	159 ± 9 B	141± 9 C	129 ± 4 B	151 ± 6 B	152 ± 10 B	153 ± 17 B	**148 C**
**Mean**	**245**	**222**	**222**	**238**	**239**	**242**	
**F-test**	16.85	24.74	48.87	15.85	11.31	11.20	**103.04**
***P*-value**	< 0.01	< 0.01	< 0.01	< 0.01	< 0.01	< 0.01	**< 0.01**
**Drought plants**							
**Fv/Fm**							
3 MAP	0.88 ± 0.01 AB	0.86 ± 0.02 AB	0.85 ± 0.02	0.87 ± 0.01 AB	0.86 ± 0.01 B	0.86 ± 0.01 AB	**0.87 B**
4 MAP	0.85 ± 0.02 AB	0.82 ± 0.01 B	0.85 ± 0.02	0.84 ± 0.02 BC	0.83 ± 0.01 B	0.83 ± 0.01 B	**0.84 C**
5 MAP	0.84 ± 0.01 AB	0.84 ± 0.01 AB	0.84 ± 0.01	0.85 ± 0.01 BC	0.85 ± 0.01 B	0.84 ± 0.01 B	**0.84 C**
6 MAP	0.89 ± 0.01 A	0.90 ± 0.02 A	0.89 ± 0.02	0.89 ± 0.01 A	0.90 ± 0.01 A	0.89 ± 0.01 A	**0.89 A**
12 MAP	0.83 ± 0.01 B	0.83 ± 0.01 B	0.83 ± 0.01	0.83 ± 0.01 C	0.83 ± 0.01 B	0.83 ± 0.01 B	**0.83 C**
**Mean**	**0.86**	**0.85**	**0.86**	**0.86**	**0.86**	**0.86**	
**F-test**	4.01	5.56	2.31	7.77	13.05	6.28	**34.56**
***P*-value**	< 0.05	< 0.01	ns	< 0.01	< 0.01	< 0.01	**< 0.01**
**ΦPSII**							
3 MAP	0.51 ± 0.03 A	0.57 ± 0.02 A	0.57 ± 0.05 A	0.51 ± 0.04 A	0.59 ± 0.03 A	0.55 ± 0.01 A	**0.55 A**
4 MAP	0.56 ± 0.03 A	0.63 ± 0.04 A	0.56 ± 0.05 A	0.63 ± 0.03 A	0.63 ± 0.04 A	0.52 ± 0.04 A	**0.59 A**
5 MAP	0.36 ± 0.02 Bab	0.34 ± 0.02 BCab	0.32 ± 0.01 Ba-c	0.36 ± 0.01 Bab	0.34 ± 0.03 Bab	0.31 ± 0.01 BCbc	**0.34 B**
6 MAP	0.35 ± 0.01 B	0.38 ± 0.02 B	0.33 ± 0.03 B	0.36 ± 0.01 Bab	0.38 ± 0.03 B	0.39 ± 0.01 B	**0.37 B**
12 MAP	0.31 ± 0.02 B	0.24 ± 0.04 C	0.25 ± 0.03 B	0.29 ± 0.03 B	0.32 ± 0.04 B	0.26 ± 0.02 C	**0.28 C**
**Mean**	**0.42**	**0.44**	**0.41**	**0.44**	**0.46**	**0.41**	
**F-test**	20.94	28.87	16.53	22.26	16.46	35.75	**119.12**
***P*-value**	< 0.01	< 0.01	< 0.01	< 0.01	< 0.01	< 0.01	**< 0.01**
**ETR**							
3 MAP	316 ± 4 A	335 ± 10 A	364 ± 34 A	333 ± 30 A	340 ± 39 A	340 ± 20 A	**338 A**
4 MAP	295 ± 27 A	346 ± 22 A	337 ± 34 A	355 ± 8 A	322 ± 21 A	284 ± 25 A	**323 A**
5 MAP	201 ± 12 B	184 ± 11 BC	187 ± 11 B	175 ± 27 B	178 ± 8 B	184 ± 4 B	**185 B**
6 MAP	209 ± 11 B	194 ± 8 B	180 ± 7 B	184 ± 6 B	188 ± 17 B	175 ± 8 B	**188 B**
12 MAP	152 ± 11 B	130 ± 2 C	148 ± 11 B	143 ± 5 B	131 ± 10 B	139 ± 5 B	**141 C**
**Mean**	**235**	**238**	**244**	**238**	**233**	**225**	
**F-test**	19.75	56.16	18.27	26.58	17.48	29.28	**147.34**
***P*-value**	< 0.01	< 0.01	< 0.01	< 0.01	< 0.01	< 0.01	**< 0.01**

Each capital letter at each plant age represents the mean of six genotypes. Different capital letters indicated significant ($p < 0.05$) differences among different plant age, whereas those among genotypes are denoted

with different lowercase letters. The significantly different ($p < 0.05$ and $p < 0.01$) between water regimes are donated * and **, respectively.

Table S3. Leaf gas exchange of cassava genotypes Rayong 9 (RY9), Rayong 72 (RY72), Kasetsart 50 (KU50), CMR38-125-77, CMR35-91-63 and CMR523-7 at the plant age of 3, 4, 5, 6, and 12 months after planting (MAP). Net photosynthesis (Pn), stomatal conductance (gs), transpiration rate (Tr), and water use efficiency (WUE) were measured in cassava growing under the control (continuous irrigation from 0MAP to 12MAP) and drought (irrigation was withheld for 60 days in the dry season during 4MAP and 5MAP then rewatered until 12MAP) treatment.

Parameter	Leaf gas exchange						Mean
	RY9	RY72	KU50	CMR38-125-77	CMR35-91-63	CM523-7	
**Control plants**							
**Pn (μmol CO_2 m^{-2} s^{-1})**							
3 MAP	21.98 ± 1.22 BC	18.35 ± 0.83 B	19.56 ± 1.92 BC	22.25 ± 1.39 B	20.37 ± 0.78 BC	18.04 ± 0.98 BC	**20.09 B**
4 MAP	28.78 ± 0.90 A	24.71 ± 1.63 A	27.51 ± 2.22 A	29.15 ± 0.66 A	25.99 ± 1.83 AB	22.97 ± 1.64 AB	**26.52 A****
5 MAP	28.32 ± 0.98 Aa	24.58 ± 1.65 Aab	26.90 ± 0.64 ABa	28.58 ± 1.13 Aa	27.91 ± 1.56 Aa	25.68 ± 0.33 Aab	**26.99 A****
6 MAP	23.70 ± 0.38 B	23.25 ± 0.45 AB	21.05 ± 1.54 A-C	22.98 ± 1.52 B	23.18 ± 0.64 AB	21.92 ± 1.03 AB	**22.68 B**
12 MAP	17.99 ± 1.44 Ca	4.50 ± 1.77 Cb	16.63 ± 1.83 Ca	4.59 ± 0.28 Cb	14.06 ± 2.37 Ca	15.81 ± 1.80 Ca	**12.26 C**
**Mean**	**24.16**	**19.08**	**22.33**	**21.52**	**22.31**	**20.89**	
**F-test**	18.61	38.80	7.59	82.16	11.85	9.67	**59.41**
***P*-value**	< 0.01	< 0.01	< 0.01	< 0.01	< 0.01	< 0.01	**< 0.01**
**gs (mol H_2O m^{-2} s^{-1})**							
3 MAP	0.24 ± 0.01 AB	0.18 ± 0.01 AB	0.21 ± 0.02 AB	0.24 ± 0.02 AB	0.22 ± 0.01 AB	0.18 ± 0.01 B	**0.21 B**
4 MAP	0.19 ± 0.01 Ba	0.13 ± 0.03 BCab	0.17 ± 0.03 ABab	0.18 ± 0.01 BCab	0.15 ± 0.03 ABab	0.13 ± 0.02 BCab	**0.16 B****
5 MAP	0.40 ± 0.09 A	0.28 ± 0.06 A	0.33 ± 0.09 A	0.40 ± 0.09 A	0.38 ± 0.11 A	0.40 ± 0.03 A	**0.37 A***
6 MAP	0.23 ± 0.02 AB	0.20 ± 0.01 AB	0.19 ± 0.03 AB	0.24 ± 0.02 AB	0.20 ± 0.02 AB	0.20 ± 0.01 B	**0.21 B**
12 MAP	0.09 ± 0.03 B	0.02 ± 0.01 C	0.07 ± 0.02 B	0.03 ± 0.01 C	0.03 ± 0.05 B	0.07 ± 0.02 C	**0.05 C**
**Mean**	**0.24**	**0.17**	**0.20**	**0.22**	**0.20**	**0.20**	
**F-test**	6.410	10.430	4.600	9.150	5.390	50.280	**49.810**
***P*-value**	< 0.01	< 0.01	< 0.05	< 0.01	< 0.01	< 0.01	**< 0.01**
**Tr (mmol H_2O m^{-2} s^{-1})**							
3 MAP	4.73 ± 0.34 A	3.79 ± 0.19 A	4.18 ± 0.34 AB	4.76 ± 0.26 A	4.55 ± 0.22 A	4.03 ± 0.21 B	**4.34 B**
4 MAP	3.51 ± 0.51 AB	2.57 ± 0.84 AB	3.35 ± 0.70 AB	3.34 ± 0.52 AB	2.84 ± 0.68 AB	2.66 ± 0.55 BC	**3.04 C****
5 MAP	5.29 ± 1.11 A	4.57 ± 0.98 A	4.46 ± 1.09 A	6.29 ± 1.61 A	5.31 ± 1.34 A	6.87 ± 0.60 A	**5.46 A**
6 MAP	4.25 ± 0.26 AB	3.93 ± 0.04 A	3.60 ± 0.44 AB	4.33 ± 0.31 A	3.84 ± 0.25 AB	4.01 ± 0.15 B	**3.99 BC**
12 MAP	1.68 ± 0.48 Bab	0.59 ± 0.05 Bb	1.37 ± 0.43 Bab	0.74 ± 0.06 Bab	0.56 ± 0.90 Bb	1.41 ± 0.25 Cab	**1.06 D**
**Mean**	**3.90**	**3.09**	**3.40**	**3.89**	**3.42**	**3.80**	
**F-test**	5.170	7.270	3.360	6.990	5.340	25.880	**41.010**
***P*-value**	< 0.01	< 0.01	< 0.05	< 0.01	< 0.01	< 0.01	**< 0.01**
**WUE (μmol CO_2 mmol H_2O^{-1})**							
3 MAP	4.71 ± 0.40	4.91 ± 0.32	4.73 ± 0.48 B	4.71 ± 0.36	4.54 ± 0.38	4.50 ± 0.27 B	**4.68 C**
4 MAP	8.61 ± 1.03	11.79 ± 2.28	8.94 ± 1.26 AB	9.27 ± 1.23	10.42 ± 1.84	9.30 ± 1.15 B	**9.72 AB**
5 MAP	6.56 ± 2.01	7.57 ± 2.17	9.63 ± 3.62 AB	6.60 ± 2.78	7.65 ± 2.63	3.83 ± 0.27 B	**6.97 BC**
6 MAP	5.75 ± 0.34	5.97 ± 0.14	6.05 ± 0.48 AB	5.59 ± 0.09	6.14 ± 0.32	5.52 ± 0.08 B	**5.84 BC**
12 MAP	7.57 ± 0.92	7.02 ± 2.00	16.80 ± 4.22 A	6.32 ± 0.68	12.91 ± 4.01	15.34 ± 2.54 A	**11.94 A**
**Mean**	**6.64**	**7.46**	**9.23**	**6.50**	**8.33**	**7.70**	
**F-test**	1.860	2.470	3.330	1.500	0.740	14.320	**5.970**
***P*-value**	ns	ns	< 0.05	ns	ns	< 0.01	**< 0.01**

Parameter	Leaf gas exchange						Mean
	RY9	RY72	KU50	CMR38-125-77	CMR35-91-63	CM523-7	
				**Drought plants**			
**Pn (μmol CO_2 m^{-2} s^{-1})**							
3 MAP	21.28 ± 0.79	21.55 ± 1.92 AB	21.60 ± 1.01 A	20.28 ± 0.88 AB	20.67 ± 0.69 AB	19.87 ± 1.00	**20.87 AB**
4 MAP	24.05 ± 2.02	21.07 ± 2.39 AB	20.43 ± 1.44 A	20.24 ± 3.37 AB	21.70 ± 1.97 A	22.41 ± 2.47	**21.65 AB**
5 MAP	19.13 ± 2.32 bc	13.66 ± 2.61 Bcd	12.79 ± 1.08 Bd	13.78 ± 1.08 Bcd	14.64 ± 0.63 Bcd	14.50 ± 2.77 cd	**14.75 C**
6 MAP	23.54 ± 0.69	23.10 ± 0.48 A	22.66 ± 0.29 A	24.41 ± 0.59 A	23.70 ± 1.22 A	23.18 ± 0.38	**23.43 A**
12 MAP	22.74 ± 1.33 a	18.26 ± 1.60 ABa	21.85 ± 0.24 Aa	17.10 ± 3.20 ABa	17.60 ± 2.00 ABa	18.74 ± 2.72 a	**19.38 B****
**Mean**	**22.15**	**19.53**	**19.87**	**19.17**	**19.66**	**19.74**	
**F-test**	1.60	3.64	18.54	3.31	6.22	2.66	**21.70**
***P*-value**	ns	< 0.05	< 0.01	< 0.05	< 0.01	ns	**< 0.01**
**gs (mol H_2O m^{-2} s^{-1})**							
3 MAP	0.26 ± 0.03 A	0.24 ± 0.05 A	0.24 ± 0.03 A	0.23 ± 0.02 A	0.24 ± 0.02 A	0.20 ± 0.01 AB	**0.23 A**
4 MAP	0.09 ± 0.01 Cab	0.08 ± 0.03 Cab	0.06 ± 0.01 Cb	0.11 ± 0.04 Bab	0.09 ± 0.03 Bab	0.11 ± 0.04 Bab	**0.09 B**
5 MAP	0.21 ± 0.02 AB	0.10 ± 0.02 C	0.09 ± 0.01 BC	0.09 ± 0.01 B	0.11 ± 0.01 B	0.12 ± 0.02 B	**0.12 B**
6 MAP	0.24 ± 0.01 A	0.22 ± 0.01 AB	0.22 ± 0.01 A	0.25 ± 0.01 A	0.22 ± 0.03 A	0.23 ± 0.01 A	**0.23 A**
12 MAP	0.14 ± 0.01 BC	0.10 ± 0.02 BC	0.14 ± 0.01 B	0.10 ± 0.03 B	0.10 ± 0.02 B	0.11 ± 0.03 B	**0.12 B**
**Mean**	**0.19**	**0.15**	**0.15**	**0.16**	**0.16**	**0.16**	
**F-test**	16.090	7.890	25.600	11.550	11.880	5.360	**55.670**
***P*-value**	< 0.01	< 0.01	< 0.01	< 0.01	< 0.01	< 0.01	**< 0.01**
**Tr (mmol H_2O m^{-2} s^{-1})**							
3 MAP	5.30 ± 0.34 A	5.17 ± 0.85 A	5.03 ± 0.52 A	4.85 ± 0.37 A	4.87 ± 0.45 A	4.33 ± 0.39 A	**4.93 A**
4 MAP	1.94 ± 0.35 B	1.93 ± 0.77 B	1.52 ± 0.51 C	2.51 ± 0.98 AB	2.06 ± 0.72 B	2.45 ± 0.91 B	**2.07 C**
5 MAP	4.71 ± 0.36 A	2.41 ± 0.42 B	2.42 ± 0.19 C	2.70 ± 0.68 AB	3.26 ± 0.60 AB	2.78 ± 0.40 AB	**3.05 B**
6 MAP	4.43 ± 0.24 A	4.241 ± 0.09 AB	4.26 ± 0.02 AB	4.48 ± 0.11 AB	4.34 ± 0.31 A	4.30 ± 0.14 A	**4.34 A**
12 MAP	2.89 ± 0.37 Ba	2.11 ± 0.36 Bab	2.81 ± 0.23 BCab	2.09 ± 0.51 Bab	2.02 ± 0.22 Bab	2.18 ± 0.54 Bab	**2.35 BC***
**Mean**	**3.86**	**3.18**	**3.21**	**3.33**	**3.32**	**3.21**	
**F-test**	17.160	6.420	16.190	4.270	6.780	3.660	**38.300**
***P*-value**	< 0.01	< 0.01	< 0.01	< 0.05	< 0.01	< 0.05	**< 0.01**
**WUE (μmol CO_2 mmol H_2O^{-1})**							
3 MAP	4.07 ± 0.33 B	4.45 ± 0.64 B	4.42 ± 0.48 B	4.27 ± 0.47	4.40 ± 0.55 B	4.73 ± 0.52	**4.39 C**
4 MAP	14.60 ± 3.12 A	16.62 ± 4.01 A	17.26 ± 4.21 A	16.25 ± 6.46	15.80 ± 5.84 A	17.06 ± 9.14	**16.27 A***
5 MAP	4.61 ± 0.75 B	5.47 ± 0.49 B	5.93 ± 0.88 B	6.15 ± 1.14	4.93 ± 0.71 B	5.20 ± 0.49	**5.38 BC**
6 MAP	5.54 ± 0.22 B	5.52 ± 0.07 B	5.46 ± 0.09 B	6.47 ± 0.87	5.51 ± 0.15 B	5.49 ± 0.15	**5.67 BC**
12 MAP	9.44 ± 2.03 AB	9.29 ± 0.94 AB	8.03 ± 0.62 B	9.05 ± 0.97	9.10 ± 0.28 AB	8.30 ± 0.44	**8.87 B**
**Mean**	**7.66**	**8.27**	**8.22**	**8.44**	**7.95**	**8.16**	
**F-test**	6.710	7.150	7.120	2.440	3.240	1.590	**24.810**
***P*-value**	< 0.01	< 0.01	< 0.01	ns	< 0.05	ns	**< 0.01**

Each capital letter at each plant age represents the mean of six genotypes. Different capital letters indicated significant ($p < 0.05$) differences among different plant age, whereas those among genotypes are denoted with different lowercase letters. The significantly different ($p < 0.05$ and $p < 0.01$) between water regimes are donated * and **, respectively.

Table S4. Leaf sugar (reducing, non-reducing and total) and starch of six cassava genotypes including Rayong 9 (RY9), Rayong 72 (RY72), Kasetsart 50 (KU50), CMR38-125-77, CMR35-91-63 and CMR523-7 at the plant age of 3, 4, 5, 6, and 12 months after planting (MAP). The plants were grown under the control (continuous irrigation from 0MAP to 12MAP) and drought (irrigation was withheld for 60 days in the dry season during 4MAP and 5MAP then rewatered until 12AMP) treatment.

Parameter	Leaf sugar content						Mean
	RY9	RY72	KU50	CMR38-125-77	CMR35-91-63	CM523-7	
**Control plants**							
**Reducing sugar of leaf (mg g^{-1})**							
3 MAP	11.94 ± 0.57 A	9.15 ± 1.89	9.45 ± 0.78	9.76 ± 1.17	10.46 ± 0.93	9.80 ± 1.89	**10.09 A**
4 MAP	10.05 ± 0.81 ABa	3.68 ± 1.00 c	5.10 ± 2.33 bc	6.44 ± 1.02 a-c	6.02 ± 0.57 a-c	6.17 ± 2.78 a-c	**6.24 B**
5 MAP	10.26 ± 0.64 AB	7.84 ± 0.55	6.62 ± 2.86	6.71 ± 1.47	7.66 ± 0.94	6.84 ± 0.52	**7.65 AB**
6 MAP	9.14 ± 1.57 AB	6.44 ± 1.90	6.50 ± 1.78	6.04 ± 1.16	6.11 ± 0.39	10.05 ± 0.99	**7.38 B**
12 MAP	6.73 ± 0.67 B	7.66 ± 2.64	11.83 ± 0.60	7.70 ± 2.18	6.49 ± 1.78	10.92 ± 3.51	**8.55 AB****
**Mean**	**9.63**	**6.96**	**7.91**	**7.34**	**7.35**	**8.76**	
**F-test**	4.260	1.380	2.070	1.030	3.200	0.890	**4.50**
***P*-value**	< 0.05	ns	ns	ns	ns	ns	**< 0.01**
**Non-reducing sugar of leaf (mg g^{-1})**							
3 MAP	15.30 ± 0.39 AB	16.11 ± 1.50	16.43 ± 0.46 AB	15.26 ± 2.38	14.16 ± 0.69	15.27 ± 1.25	**15.42 AB**
4 MAP	9.78 ± 0.87 C	14.15 ± 1.31	14.02 ± 1.17 AB	9.56 ± 1.71	11.60 ± 0.66	10.96 ± 0.38	**11.68 BC**
5 MAP	11.02 ± 0.06 BC	9.82 ± 1.41	8.60 ± 2.88 B	11.05 ± 2.26	10.84 ± 1.97	9.31 ± 1.13	**10.10 C**
6 MAP	18.00 ± 1.44 Aab	19.39 ± 1.82 ab	19.76 ± 2.11 Aab	16.27 ± 5.84 ab	17.66 ± 1.23 ab	13.83 ± 1.42 ab	**17.49 A**
12 MAP	14.63 ± 1.79 A-C	20.60 ± 5.80	11.59 ± 1.84 AB	16.53 ± 3.44	17.45 ± 4.53	15.83 ± 5.29	**16.10 A**
**Mean**	**13.75**	**16.02**	**14.08**	**13.74**	**14.35**	**13.04**	
**F-test**	8.480	2.170	5.220	0.860	1.890	1.200	**10.58**
***P*-value**	< 0.01	ns	< 0.05	ns	ns	ns	**< 0.01**
**Total sugar of leaf (mg g^{-1})**							
3 MAP	27.25 ± 0.39 A	25.27 ± 0.77 AB	25.89 ± 0.89	25.02 ± 1.22	24.63 ± 1.29	25.07 ± 0.90 A	**25.52 A**
4 MAP	19.84 ± 1.58 B	17.83 ± 0.31 B	19.12 ± 2.96	16.01 ± 0.91	17.63 ± 0.11	17.13 ± 2.45 B	**17.93 B**
5 MAP	21.28 ± 1.16 AB	17.66 ± 1.63 B	15.22 ± 5.74	17.77 ± 1.28	18.50 ± 1.61	16.15 ± 1.64 B	**17.76 B**
6 MAP	27.14 ± 1.11 Aab	25.84 ± 2.85 ABab	26.27 ± 2.11 ab	22.32 ± 5.80 bc	23.77 ± 1.47 abc	23.89 ± 0.53 Aba-c	**24.87 A**
12 MAP	21.36 ± 2.13 AB	28.26 ± 3.39 A	23.43 ± 2.06	24.24 ± 2.89	23.95 ± 3.57	26.75 ± 2.18 A	**24.66 A**
**Mean**	**23.38**	**22.98**	**21.99**	**21.08**	**21.70**	**21.80**	
**F-test**	6.440	5.230	2.190	1.740	2.920	8.020	**19.34**
***P*-value**	< 0.01	< 0.05	ns	ns	ns	< 0.01	**< 0.01**
**Starch of leaf (mg g^{-1})**							
3 MAP	1.50 ± 0.20 cd	1.30 ± 0.15 d	1.76 ± 0.09 bc	2.02 ± 0.10 Aab	1.63 ± 0.13 a-d	1.80 ± 0.07 Aa-c	**1.67 A**
4 MAP	1.53 ± 0.11	0.94 ± 0.17	0.66 ± 0.27	1.19 ± 0.15 B	1.20 ± 0.56	1.21 ± 0.21 AB	**1.12 B**
5 MAP	1.38 ± 0.02	0.81 ± 0.20	0.79 ± 0.04	1.10 ± 0.23 B	1.09 ± 0.51	1.12 ± 0.28 AB	**1.05 B**
6 MAP	1.37 ± 0.21	1.03 ± 0.28	1.32 ± 0.45	1.33 ± 0.19 AB	1.44 ± 0.13	1.57 ± 0.16 AB	**1.34 AB**
12 MAP	1.42 ± 0.34 a	1.31 ± 0.09 ab	0.93 ± 0.08 bc	0.83 ± 0.09 Bbc	0.73 ± 0.07 c	0.97 ± 0.08 Ba-c	**1.03 B**
**Mean**	**1.44**	**1.08**	**1.10**	**1.30**	**1.22**	**1.34**	
**F-test**	0.760	1.430	3.440	7.620	0.970	3.750	**7.86**
***P*-value**	ns	ns	ns	< 0.01	ns	< 0.05	**< 0.01**
**Drought plants**							
**Reducing sugar of leaf (mg g^{-1})**							
3 MAP	10.17 ± 1.32	10.79 ± 3.44	10.77 ± 2.11	13.51 ± 2.70 A	7.44 ± 3.02	9.78 ± 3.14	**10.41 A**
4 MAP	9.74 ± 1.32 a	9.27 ± 1.97 ab	4.66 ± 0.99 c	6.14 ± 1.88 Aba-c	4.33 ± 0.94 c	6.70 ± 1.78 a-c	**6.81 B**
5 MAP	11.15 ± 3.49	7.74 ± 0.85	5.31 ± 0.80	8.92 ± 0.85 AB	7.54 ± 2.32	6.10 ± 0.56	**7.79 AB**
6 MAP	9.89 ± 1.24	7.13 ± 2.79	5.59 ± 1.15	5.49 ± 1.00 B	8.79 ± 2.83	6.03 ± 1.40	**7.15 B**
12 MAP	11.58 ± 0.11	6.85 ± 2.30	8.59 ± 1.72	4.59 ± 1.29 B	6.34 ± 1.67	6.31 ± 1.36	**7.38 AB**
**Mean**	**10.51**	**8.36**	**6.99**	**7.74**	**6.89**	**6.99**	
**F-test**	0.190	0.460	3.250	4.580	0.530	0.730	**3.11**

Parameter	Leaf sugar content						Mean
	RY9	RY72	KU50	CMR38-125-77	CMR35-91-63	CM523-7	
***P*-value**	ns	ns	ns	< 0.05	ns	ns	**< 0.05**
**Non-reducing sugar of leaf (mg g^{-1})**							
3 MAP	16.62 ± 2.90 A	15.28 ± 2.24	15.67 ± 2.33 AB	14.42 ± 3.33	13.29 ± 0.86	17.80 ± 3.70	**15.51 ABC**
4 MAP	8.67 ± 1.35 B	12.69 ± 3.55	13.38 ± 2.56 B	11.88 ± 2.59	14.15 ± 3.26	11.13 ± 1.54	**11.98 C**
5 MAP	18.55 ± 0.61 A	16.38 ± 3.10	18.89 ± 3.39 AB	16.72 ± 3.11	18.24 ± 5.29	9.74 ± 2.08	**16.42 AB**
6 MAP	18.13 ± 0.81 Aab	17.97 ± 2.19 ab	24.02 ± 2.84 Aa	20.58 ± 2.14 a	20.34 ± 1.56 a	12.48 ± 2.55 b	**18.92 A**
12 MAP	12.61 ± 0.95 AB	14.77 ± 3.08	11.16 ± 0.64 B	16.06 ± 2.54	17.55 ± 3.30	15.19 ± 1.67	**14.56 BC**
**Mean**	**14.92**	**15.42**	**16.63**	**15.94**	**16.72**	**13.27**	
**F-test**	7.270	0.460	3.520	1.330	0.820	1.760	**5.37**
***P*-value**	< 0.01	ns	< 0.05	ns	ns	ns	**< 0.01**
**Total sugar of leaf (mg g^{-1})**							
3 MAP	26.79 ± 1.59 AB	26.07 ± 1.23	26.44 ± 0.23 AB	27.93 ± 0.91 A	20.73 ± 3.38	27.59 ± 1.34	**25.930 A**
4 MAP	18.42 ± 1.97 B	21.96 ± 3.56	18.04 ± 1.72 B	18.02 ± 3.26 C	18.49 ± 2.73	17.84 ± 1.70	**18.80 B**
5 MAP	29.70 ± 3.91 A	24.13 ± 3.77	24.20 ± 4.22 AB	25.64 ± 2.99 AB	25.792 ± 5.23	15.84 ± 2.65	**24.22 A**
6 MAP	28.031 ± 1.04 ABab	25.10 ± 1.54 a-c	29.62 ± 1.70 Aa	26.08 ± 1.30 ABab	29.13 ± 1.59 ab	18.52 ± 1.38 c	**26.08 A**
12 MAP	24.19 ± 0.84 AB	21.63 ± 0.94	19.75 ± 1.53 AB	20.66 ± 1.30 BC	23.89 ± 2.19	21.50 ± 0.57	**21.94 AB**
**Mean**	**25.43**	**23.78**	**23.62**	**23.67**	**23.61**	**20.26**	
**F-test**	4.140	0.590	4.340	3.610	1.630	7.510	**8.04**
***P*-value**	< 0.05	ns	< 0.05	< 0.05	ns	< 0.01	**< 0.01**
**Starch of leaf (%)**							
3 MAP	2.20 ± 0.02 Aa	1.44 ± 0.19 cd	1.66 ± 0.12 Ab-d	1.76 ± 0.10 Abc	1.41 ± 0.19 ABcd	1.81 ± 0.05 a-c	**1.71 A**
4 MAP	0.53 ± 0.08 C	0.80 ± 0.25	0.51 ± 0.04 B	0.65 ± 0.22 B	0.68 ± 0.16 C	0.51 ± 0.02	**0.61 B**
5 MAP	0.76 ± 0.11 BC	0.59 ± 0.11	0.66 ± 0.05 B	0.52 ± 0.06 B	0.65 ± 0.16 C	0.41 ± 0.04	**0.60 B**
6 MAP	1.47 ± 0.21 AB	1.48 ± 0.45	1.40 ± 0.18 A	1.80 ± 0.17 A	1.72 ± 0.18 A	1.32 ± 0.12	**1.53 A**
12 MAP	1.27 ± 0.32 BCab	0.91 ± 0.18 bc	0.85 ± 0.12 Bbc	0.72 ± 0.03 Bc	0.85 ± 0.04 BCbc	0.68 ± 0.04 c	**0.88 B**
**Mean**	**1.25**	**1.05**	**1.02**	**1.09**	**1.07**	**0.95**	
**F-test**	12.890	2.280	18.430	22.120	9.390	82.850	**54.00**
***P*-value**	< 0.01	ns	< 0.01	< 0.01	< 0.01	< 0.01	**< 0.01**

Each capital letter at each plant age represents the mean of six genotypes. Different capital letters indicated significant ($p < 0.05$) differences among different plant age, whereas those among genotypes are denoted with different lowercase letters. The significantly different ($p < 0.05$ and $p < 0.01$) between water regimes are donated * and **, respectively.

Table S5. Stem sugar (reducing, non-reducing and total) and starch of six cassava genotypes including Rayong 9 (RY9), Rayong 72 (RY72), Kasetsart 50 (KU50), CMR38-125-77, CMR35-91-63 and CMR523-7 at the plant age of 3, 4, 5, 6, and 12 months after planting (MAP). The plants were grown under the control (continuous irrigation from 0MAP to 12MAP) and drought (irrigation was withheld for 60 days in the dry season during 4MAP and 5MAP then rewatered until 12AMP) treatment.

Parameter	Stem sugar content						Mean
	**RY9**	**RY72**	**KU50**	**CMR38-125-77**	**CMR35-91-63**	**CM523-7**	
			**Control plants**				
**Reducing sugar of stem (mg g^{-1})**							
3 MAP	0.93 ± 0.55 Bb	1.10 ± 0.38 b	0.90 ± 0.23 b	0.86 ± 0.02 b	1.52 ± 0.16 b	1.18 ± 0.20 Bb	**1.08 B**
4 MAP	2.91 ± 0.87 AB	2.09 ± 1.43	1.00 ± 0.29	2.52 ± 1.02	3.61 ± 1.58	1.96 ± 0.37 B	**2.35 B**
5 MAP	2.87 ± 0.97 AB	1.28 ± 0.25	2.71 ± 0.24	2.57 ± 1.25	2.24 ± 0.23	1.44 ± 0.32 B	**2.18 B***
6 MAP	2.09 ± 0.78 B	3.10 ± 0.97	4.31 ± 3.50	4.16 ± 1.07	2.83 ± 1.10	1.71 ± 0.62 B	**3.03 AB**
12 MAP	6.54 ± 1.36 Ab	2.63 ± 1.40 c	1.62 ± 1.02 c	2.12 ± 0.44 c	4.67 ± 1.08 bc	11.17 ± 3.01 Aa	**4.79 A***
**Mean**	**3.07**	**2.04**	**2.11**	**2.45**	**2.98**	**3.49**	
**F-test**	4.950	0.710	0.760	1.760	1.500	9.490	**5.92**
***P*-value**	< 0.05	ns	ns	ns	ns	< 0.01	**< 0.01**
**Non-reducing sugar of stem (mg g^{-1})**							
3 MAP	5.83 ± 1.62 ABabc	7.61 ± 0.22 Ba	7.66 ± 0.42 a	6.47 ± 0.59 BCab	4.40 ± 0.46 BCbc	6.46 ± 1.09 ab	**6.41 B***
4 MAP	1.41 ± 0.47 B	3.04 ± 0.51 B	3.93 ± 1.86	2.62 ± 0.03 C	1.67 ± 0.15 C	4.41 ± 3.02	**2.85 C**
5 MAP	6.16 ± 1.53 AB	4.61 ± 2.05 B	4.69 ± 0.77	4.75 ± 1.84 BC	4.33 ± 0.72 BC	5.71 ± 1.85	**5.04 BC**
6 MAP	5.78 ± 2.43 AB	6.29 ± 0.89 B	9.67 ± 4.11	9.13 ± 0.83 AB	6.18 ± 0.91 B	5.80 ± 1.78	**7.14 B**
12 MAP	8.83 ± 0.57 A	12.97 ± 1.01 A	12.96 ± 0.97	12.47 ± 1.25 A	10.44 ± 1.26 A	8.69 ± 1.61	**11.06 A**
**Mean**	**5.61**	**6.91**	**7.79**	**7.09**	**5.41**	**6.22**	
**F-test**	3.110	11.460	3.110	12.310	16.570	0.630	**24.64**
***P*-value**	ns	< 0.01	ns	< 0.01	< 0.01	ns	**< 0.01**
**Total sugar of stem (mg g^{-1})**							
3 MAP	6.77 ± 1.87 b-d	8.72 ± 0.57 ABab	8.56 ± 0.28 Ba-c	7.33 ± 0.58 ABa-d	5.93 ± 0.30 Bcd	7.65 ± 1.18 Ba-d	**7.49 BC**
4 MAP	4.32 ± 1.34	5.13 ± 1.91 B	4.93 ± 1.75 B	5.14 ± 1.00 B	5.29 ± 1.48 B	6.37 ± 3.10 B	**5.20 C**
5 MAP	9.04 ± 2.36	5.90 ± 2.19 B	7.40 ± 0.99 B	7.32 ± 3.03 AB	6.57 ± 0.70 B	7.15 ± 2.13 B	**7.23 C**
6 MAP	7.88 ± 3.20	9.39 ± 1.37 AB	13.99 ± 1.08 A	13.29 ± 1.06 A	9.01 ± 1.63 B	7.51 ± 2.15 B	**10.18 B**
12 MAP	15.38 ± 1.88	15.61 ± 2.38 A	14.58 ± 1.01 A	14.59 ± 1.11 A	15.11 ± 1.25 A	19.87 ± 2.01 A	**15.86 A**
**Mean**	**8.68**	**8.95**	**9.90**	**9.54**	**8.38**	**9.71**	
**F-test**	3.470	5.250	14.170	6.690	11.600	6.680	**34.07**
***P*-value**	ns	< 0.05	< 0.01	< 0.01	< 0.01	< 0.01	**< 0.01**
**Starch of stem (%)**							
3 MAP	2.11 ± 0.38 Bb	1.37 ± 0.33 Bb	2.19 ± 0.19 BCb	4.34 ± 1.42 ABa	0.76 ± 0.14 Bb	2.17 ± 0.51 b	**2.16 B**
4 MAP	1.48 ± 0.34 B	0.80 ± 0.12 B	1.24 ± 0.41 C	1.65 ± 0.23 B	2.14 ± 0.75 B	0.76 ± 0.07	**1.34 B**
5 MAP	1.29 ± 0.25 B	1.64 ± 0.28 B	2.44 ± 0.28 B	2.10 ± 1.03 B	2.46 ± 0.65 B	1.94 ± 0.70	**1.98 B**
6 MAP	1.05 ± 0.06 B	1.05 ± 0.09 B	1.23 ± 0.10 C	1.18 ± 0.17 B	1.39 ± 0.24 B	1.22 ± 0.08	**1.19 B**
12 MAP	5.81 ± 0.77 Aabc	6.55 ± 0.37 Aab	4.51 ± 0.18 Acd	7.67 ± 1.00 Aa	6.65 ± 0.28 Aab	2.11 ± 0.53 e	**5.55 A**
**Mean**	**2.35**	**2.28**	**2.33**	**3.39**	**2.68**	**1.65**	
**F-test**	21.360	83.350	28.190	8.650	23.420	1.830	**35.80**
***P*-value**	< 0.01	< 0.01	< 0.01	< 0.01	< 0.01	ns	**< 0.01**
			**Drought plants**				
**Reducing sugar of stem (mg g^{-1})**							
3 MAP	0.61 ± 0.28 Bb	0.70 ± 0.15 b	2.10 ± 1.12 b	1.13 ± 0.31 b	1.12 ± 0.28 b	5.38 ± 2.10 a	**1.84 AB**
4 MAP	1.00 ± 0.46 B	1.31 ± 0.24	3.40 ± 1.10	2.36 ± 0.42	1.56 ± 0.92	1.91 ± 0.49	**1.93 AB**
5 MAP	1.79 ± 0.61 AB	2.01 ± 0.22	1.02 ± 0.34	1.09 ± 0.27	1.50 ± 0.09	1.49 ± 0.11	**1.48 B**
6 MAP	3.38 ± 0.80 A	3.17 ± 1.23	2.85 ± 0.81	2.41 ± 0.92	2.43 ± 0.83	4.94 ± 2.47	**3.20 A**
12 MAP	3.36 ± 0.90 Abc	3.17 ± 1.77 bc	2.22 ± 0.86 c	2.40 ± 1.16 c	2.29 ± 1.29 c	4.84 ± 1.43 bc	**3.04 AB**
**Mean**	**2.03**	**2.08**	**2.32**	**1.88**	**1.78**	**3.72**	
**F-test**	3.940	1.270	1.010	0.960	0.480	1.340	**3.40**
***P*-value**	< 0.05	ns	ns	ns	ns	ns	**< 0.05**
**Non-reducing sugar of stem (mg g^{-1})**							
3 MAP	4.02 ± 0.47 Bc	4.51 ± 1.06 bc	7.91 ± 0.81 ABa	4.13 ± 0.34 Bc	5.28 ± 0.74 ABbc	4.74 ± 0.83 Bbc	**5.10 B**

Parameter	Stem sugar content						Mean
	RY9	RY72	KU50	CMR38-125-77	CMR35-91-63	CM523-7	
4 MAP	1.51 ± 0.46 B	4.17 ± 1.17	3.03 ± 1.45 BC	1.93 ± 0.65 B	3.06 ± 0.48 B	1.71 ± 0.54 B	**2.57 C**
5 MAP	2.79 ± 0.62 B	4.69 ± 0.86	1.40 ± 0.91 C	1.89 ± 0.77 B	2.60 ± 1.41 B	4.32 ± 0.08 B	**2.95 C**
6 MAP	4.41 ± 0.43 B	6.54 ± 2.13	5.51 ± 1.96 ABC	5.59 ± 1.19 B	5.48 ± 1.83 AB	5.68 ± 0.32 B	**5.53 B**
12 MAP	11.18 ± 1.39 A	12.06 ± 3.00	11.42 ± 1.18 A	9.57 ± 0.89 A	11.64 ± 3.20 A	11.15 ± 2.05 A	**11.17 A**
**Mean**	**4.79**	**6.40**	**5.86**	**4.63**	**5.62**	**5.53**	
**F-test**	24.000	3.250	8.930	15.160	3.980	11.430	**44.05**
***P*-value**	< 0.01	ns	< 0.01	< 0.01	< 0.05	< 0.01	**< 0.01**
**Total sugar of stem (mg g^{-1})**							
3 MAP	4.64 ± 0.56 BCd	5.22 ± 0.92 d	10.02 ± 1.57 ABa	5.27 ± 0.09 Bd	6.40 ± 0.68 a-d	10.12 ± 1.51 ABa	**6.94 BC**
4 MAP	2.52 ± 0.16 C	5.49 ± 0.96	6.44 ± 0.71 AB	4.30 ± 0.44 B	4.62 ± 0.96	3.62 ± 0.85 B	**4.50 C**
5 MAP	4.59 ± 1.14 BC	6.70 ± 0.67	2.42 ± 1.24 B	2.98 ± 0.72 B	4.10 ± 1.38	5.81 ± 0.19 B	**4.43 C**
6 MAP	7.79 ± 0.94 B	9.71 ± 3.35	8.37 ± 2.46 AB	8.01 ± 1.77 AB	7.91 ± 2.59	10.62 ± 2.78 AB	**8.74 B**
12 MAP	14.55 ± 0.51 A	15.24 ± 4.43	13.65 ± 1.92 A	11.97 ± 1.99 A	13.94 ± 3.76	16.00 ± 1.27 A	**14.22 A**
**Mean**	**6.82**	**8.48**	**8.18**	**6.51**	**7.40**	**9.24**	
**F-test**	40.060	2.640	6.100	8.120	3.240	9.270	**32.01**
***P*-value**	< 0.01	ns	< 0.01	< 0.01	ns	< 0.01	**< 0.01**
**Starch of stem (%)**							
3 MAP	1.74 ± 0.21 b	1.40 ± 0.34 Bb	1.98 ± 0.25 ABb	1.62 ± 0.54 Bb	1.11 ± 0.17 Bb	1.87 ± 0.70 b	**1.62 BC**
4 MAP	0.87 ± 0.69	3.02 ± 0.98 AB	1.76 ± 0.20 AB	1.62 ± 0.34 B	1.27 ± 0.33 B	0.98 ± 0.23	**1.59 BC**
5 MAP	1.13 ± 0.37	1.95 ± 0.98 B	3.11 ± 0.79 A	4.08 ± 1.18 AB	1.77 ± 0.48 B	1.34 ± 0.09	**2.23 B**
6 MAP	0.40 ± 0.05	0.81 ± 0.06 B	0.86 ± 0.30 B	0.92 ± 0.03 B	0.77 ± 0.27 B	0.91 ± 0.31	**0.78 C**
12 MAP	3.26 ± 1.08 de	5.69 ± 0.80 Abc	3.63 ± 0.10 Ade	5.60 ± 1.08 Abc	6.29 ± 0.60 Aabc	2.18 ± 0.75 e	**4.44 A**
**Mean**	**1.48**	**2.58**	**2.28**	**2.77**	**2.25**	**1.46**	
**F-test**	3.360	6.880	7.330	6.670	32.600	1.260	**22.20**
***P*-value**	ns	< 0.01	< 0.01	< 0.01	< 0.01	ns	**< 0.01**

Each capital letter at each plant age represents the mean of six genotypes. Different capital letters indicated significant ($p < 0.05$) differences among different plant age, whereas those among genotypes are denoted with different lowercase letters. The significantly different ($p < 0.05$ and $p < 0.01$) between water regimes are donated * and **, respectively.

Table S6. Tuber sugar (reducing, non-reducing and total) and starch of six cassava genotypes including Rayong 9 (RY9), Rayong 72 (RY72), Kasetsart 50 (KU50), CMR38-125-77, CMR35-91-63 and CMR523-7 at the plant age of 3, 4, 5, 6, and 12 months after planting (MAP). The plants were grown under the control (continuous irrigation from 0MAP to 12MAP) and drought (irrigation was withheld for 60 days in the dry season during 4MAP and 5MAP then rewatered until 12AMP) treatment.

Parameter	Tuber sugar						Mean
	RY9	RY72	KU50	CMR38-125-77	CMR35-91-63	CM523-7	
			**Control plants**				
**Reducing sugar of tuber (mg g^{-1})**							
3 MAP	8.66 ± 3.17	10.04 ± 3.97	10.57 ± 1.34 A	6.46 ± 2.64	7.26 ± 1.47	5.37 ± 0.30	**8.06 A**
4 MAP	4.06 ± 1.05	4.58 ± 2.00	1.95 ± 0.56 B	4.27 ± 1.75	4.88 ± 1.20	3.07 ± 0.93	**3.80 BC**
5 MAP	1.74 ± 0.15	1.02 ± 0.13	1.94 ± 0.77 B	1.23 ± 0.09	1.61 ± 0.07	0.71 ± 0.06	**1.38 C**
6 MAP	3.48 ± 1.38	5.28 ± 0.74	3.76 ± 1.58 B	3.26 ± 0.60	6.31 ± 0.44	5.10 ± 2.40	**4.53 B**
12 MAP	2.98 ± 0.77	1.63 ± 0.17	3.91 ± 1.87 B	4.18 ± 0.76	8.60 ± 2.84	3.34 ± 1.94	**4.11 B**
**Mean**	**4.19**	**4.52**	**4.43**	**3.89**	**5.74**	**3.52**	
**F-test**	2.550	3.170	7.270	1.630	3.010	1.680	**13.57**
***P*-value**	ns	ns	< 0.01	ns	ns	ns	**< 0.01**
**Non-reducing sugar of tuber (mg g^{-1})**							
3 MAP	8.26 ± 1.29 C	8.51 ± 2.05 B	10.88 ± 1.24 B	9.00 ± 1.20	9.99 ± 2.00	11.57 ± 1.65 AB	**9.70 B**
4 MAP	15.91 ± 0.54 AB	14.35 ± 1.41 B	13.17 ± 0.21 AB	13.57 ± 0.53	15.25 ± 0.73	14.70 ± 1.33 A	**14.49 A**
5 MAP	9.28 ± 0.14 BC	7.85 ± 2.05 B	9.60 ± 0.76 B	9.32 ± 0.64	9.04 ± 2.39	10.76 ± 1.84 AB	**9.31 B**
6 MAP	10.26 ± 1.25 BC	8.74 ± 1.18 B	11.07 ± 0.66 B	9.54 ± 1.35	9.45 ± 0.52	7.27 ± 1.08 B	**9.39 B**
12 MAP	17.95 ± 2.58 Ab	25.29 ± 0.91 Aa	17.08 ± 1.55 Abc	7.76 ± 2.78 e	10.50 ± 3.64 de	16.66 ± 1.28 Abc	**15.87 A**
**Mean**	**12.33**	**12.95**	**12.37**	**9.84**	**10.85**	**12.20**	
**F-test**	9.150	21.550	8.600	2.060	1.340	6.190	**14.07**
***P*-value**	< 0.01	< 0.01	< 0.01	ns	ns	< 0.01	**< 0.01**
**Total sugar of tuber (mg g^{-1})**							
3 MAP	16.92 ± 4.26	18.55 ± 5.92 AB	21.45 ± 1.74 A	15.46 ± 3.77	17.25 ± 3.37	16.94 ± 1.53 AB	**17.76 AB**
4 MAP	19.97 ± 0.64	18.93 ± 2.50 AB	15.13 ± 0.36 BC	17.85 ± 1.30	20.13 ± 0.80	17.78 ± 1.47 AB	**18.30 A**
5 MAP	11.02 ± 0.19	8.88 ± 2.13 B	11.55 ± 1.51 C	10.55 ± 0.67	10.66 ± 2.35	11.48 ± 1.81 B	**10.69 C**
6 MAP	13.74 ± 2.53	14.03 ± 1.05 AB	14.84 ± 1.90 BC	12.81 ± 0.80	15.76 ± 0.65	12.38 ± 2.24 AB	**13.93 BC**
12 MAP	20.93 ± 3.24 ab	26.93 ± 1.03 Aa	21.00 ± 0.41 ABab	11.95 ± 2.05 d	19.10 ± 4.93 bc	20.01 ± 1.68 Abc	**19.99 A**
**Mean**	**16.52**	**17.47**	**16.80**	**13.73**	**16.59**	**15.72**	
**F-test**	2.450	4.640	9.990	2.010	1.630	4.260	**14.18**
***P*-value**	ns	< 0.05	< 0.01	ns	ns	< 0.05	**< 0.01**
**Starch of tuber (%)**							
3 MAP	9.06 ± 3.53	7.33 ± 0.60 C	8.82 ± 1.34 B	10.94 ± 2.51	8.29 ± 1.27 B	7.42 ± 1.59	**8.65 D**
4 MAP	16.71 ± 1.07	15.29 ± 1.56 BC	16.02 ± 1.41 AB	14.63 ± 0.69	16.56 ± 2.00 AB	13.40 ± 0.41	**15.44 C***
5 MAP	21.71 ± 3.27	25.52 ± 3.64 A	17.39 ± 2.49 AB	27.32 ± 9.95	25.58 ± 4.86 A	20.24 ± 5.56	**22.96 A**
6 MAP	23.25 ± 4.53	19.72 ± 2.57 AB	20.46 ± 3.73 A	21.65 ± 3.02	20.58 ± 2.99 AB	20.99 ± 1.92	**21.11 AB**
12 MAP	18.30 ± 0.63 abc	17.10 ± 0.51 ABCabc	11.03 ± 1.94 ABd	21.72 ± 1.04 a	17.90 ± 2.48 ABabc	12.67 ± 3.91 cd	**16.46 BC**
**Mean**	**17.81**	**17.00**	**14.75**	**19.26**	**17.79**	**14.95**	
**F-test**	3.390	9.630	4.070	1.810	4.510	3.060	**20.12**
***P*-value**	ns	< 0.01	ns	ns	< 0.05	ns	**< 0.01**
			**Drought plants**				
**Reducing sugar of tuber (mg g^{-1})**							
3 MAP	9.75 ± 1.32 A	6.15 ± 1.32	8.13 ± 0.47 A	6.36 ± 0.58	12.90 ± 0.36	7.76 ± 0.80	**8.51 A**
4 MAP	2.39 ± 0.53 B	3.48 ± 1.33	4.56 ± 2.19 AB	2.90 ± 0.35	5.98 ± 2.86	4.86 ± 2.07	**4.03 B**
5 MAP	4.21 ± 1.63 B	3.45 ± 1.09	2.42 ± 0.53 B	1.99 ± 0.63	3.12 ± 0.96	2.21 ± 1.46	**2.90 B**
6 MAP	4.19 ± 1.11 B	3.51 ± 2.29	2.28 ± 0.53 B	2.64 ± 0.49	2.17 ± 0.98	4.98 ± 0.79	**3.29 B**
12 MAP	2.24 ± 0.70 B	2.96 ± 1.29	3.51 ± 1.32 AB	3.24 ± 2.35	5.22 ± 2.69	2.98 ± 2.03	**3.36 B**
**Mean**	**4.56**	**3.92**	**4.19**	**3.43**	**5.88**	**4.56**	
**F-test**	7.230	0.700	3.910	2.190	5.110	1.970	**15.46**
***P*-value**	< 0.01	ns	< 0.05	ns	< 0.05	ns	**< 0.01**
**Non-reducing sugar of tuber (mg g^{-1})**							
3 MAP	15.09 ± 1.76	8.78 ± 1.83 B	10.97 ± 1.11 BC	9.75 ± 0.75	11.27 ± 0.75	10.37 ± 1.85 AB	**11.04 BC**

Parameter	Tuber sugar						Mean
	**RY9**	**RY72**	**KU50**	**CMR38-125-77**	**CMR35-91-63**	**CM523-7**	
4 MAP	13.14 ± 1.48	13.19 ± 0.53 AB	12.99 ± 1.15 AB	11.32 ± 0.61	12.78 ± 2.02	9.83 ± 2.29 AB	**12.21 B**
5 MAP	9.04 ± 1.37	11.52 ± 0.03 AB	7.88 ± 0.81 C	8.02 ± 0.87	8.80 ± 0.94	9.25 ± 1.80 AB	**9.08 CD**
6 MAP	11.14 ± 2.59	6.76 ± 1.95 B	8.74 ± 0.99 BC	6.65 ± 1.06	8.73 ± 0.96	6.83 ± 1.33 B	**8.14 D**
12 MAP	17.05 ± 2.04 bc	17.87 ± 1.56 Ab	15.85 ± 0.23 Abc	9.98 ± 2.99 de	13.1 ± 1.61 cd	17.21 ± 1.53 Abc	**15.18 A**
**Mean**	**13.10**	**11.63**	**11.29**	**9.15**	**10.95**	**10.70**	
**F-test**	2.760	9.280	12.280	1.420	2.450	4.710	**16.22**
***P*-value**	ns	< 0.01	< 0.01	ns	ns	< 0.05	**< 0.01**
**Total sugar of tuber (mg g^{-1})**							
3 MAP	24.84 ± 0.48 A	14.93 ± 2.49 AB	19.11 ± 1.52 A	16.12 ± 0.24 A	24.18 ± 1.08 A	18.14 ± 2.56	**19.55 A**
4 MAP	15.54 ± 0.96 B	16.68 ± 1.74 AB	17.55 ± 1.25 A	14.22 ± 0.34 AB	18.76 ± 1.79 AB	14.70 ± 0.48	**16.24 A**
5 MAP	13.25 ± 2.65 B	14.97 ± 1.09 AB	10.31 ± 1.34 B	10.02 ± 1.22 BC	11.92 ± 1.81 B	11.46 ± 3.22	**11.99 B**
6 MAP	15.33 ± 1.92 B	10.28 ± 1.50 B	11.02 ± 1.03 B	9.29 ± 1.25 C	10.90 ± 1.83 B	11.82 ± 2.02	**11.44 B**
12 MAP	19.29 ± 2.07 ABbc	20.84 ± 2.59 Aab	19.36 ± 1.14 Abc	13.22 ± 1.07 ABCcd	18.36 ± 3.35 ABb-d	20.19 ± 3.56 ab	**18.54 A**
**Mean**	**17.66**	**15.55**	**15.47**	**12.58**	**16.83**	**15.26**	
**F-test**	6.480	3.730	12.300	9.490	6.720	2.180	**18.25**
***P*-value**	< 0.01	< 0.05	< 0.01	< 0.01	< 0.01	ns	**< 0.01**
**Starch of tuber (%)**							
3 MAP	10.40 ± 2.02	11.43 ± 0.99 B	10.37 ± 2.27 B	11.84 ± 1.83 B	8.07 ± 1.89 C	10.17 ± 2.01	**10.38 B**
4 MAP	14.48 ± 0.46	14.10 ± 2.85 AB	15.33 ± 1.88 AB	17.46 ± 1.22 AB	11.17 ± 1.85 BC	10.00 ± 1.15	**13.76 B**
5 MAP	18.70 ± 4.45	22.24 ± 3.73 A	22.76 ± 2.99 A	20.56 ± 3.52 AB	19.54 ± 0.92 A	11.87 ± 0.85	**19.28 A**
6 MAP	18.83 ± 3.60	24.51 ± 1.34 A	22.04 ± 0.79 A	21.26 ± 0.63 A	15.40 ± 1.89 ABC	15.84 ± 1.54	**19.64 A**
12 MAP	21.51 ± 1.30 a	18.80 ± 0.80 ABab	18.70 ± 1.96 Abab	22.21 ± 1.37 Aa	16.78 ± 1.70 ABa-c	14.84 ± 2.5 b-d	**18.81 A***
**Mean**	**16.79**	**18.22**	**17.85**	**18.67**	**14.20**	**12.55**	
**F-test**	2.460	5.840	5.920	4.550	7.290	2.440	**17.40**
***P*-value**	ns	< 0.05	< 0.05	< 0.05	< 0.01	ns	**< 0.01**

Each capital letter at each plant age represents the mean of six genotypes. Different capital letters indicated significant ($p < 0.05$) differences among different plant age, whereas those among genotypes are denoted with different lowercase letters. The significantly different ($p < 0.05$ and $p < 0.01$) between water regimes are donated * and **, respectively.

Table S7. Leaf, petiole, stem, root, tuber, total biomass, partitioning index, and leaf are index of six cassava genotypes including Rayong9 (RY9), Rayong72 (RY72), Kasetsart50 (KU50), CMR38-125-77, CMR35-91-63 and CMR523-7 at the plant age of 3, 4, 5, 6 and 12 months after planting (MAP). The plants were grown under the control (continuous irrigation from 0MAP to 12MAP) and drought (irrigation was withheld for 60 days in the dry season during 4MAP and 5MAP then rewatered until 12MAP) treatment. Each capital letter at each plant age represents the mean of six genotypes. [Partitioning index (PI) is the ratio between tuber dry weight and total dry weight. Leaf area index (LAI) was determined by using Line Quantum Sensor and calculated according to Santanoo et al. (2020)]

Parameter	Plant biomass of cassava						Mean
	**RY9**	**RY72**	**KU50**	**CMR38-125-77**	**CMR35-91-63**	**CM523-7**	
			**Control plants**				
**Leaf dry weight (g plant^{-1})**							
3 MAP	16.9 ± 2.1 B	16.0 ± 5.6	20.9 ± 5.1 C	19.0 ± 4.5 B	19.0 ± 4.5 B	15.3 ± 2.3 C	**17.8 D**
4 MAP	41.1 ± 4.7 B	36.0 ± 7.8	62.3 ± 10.5 B	46.8 ± 9.1 B	45.5 ± 6.7 AB	49.7 ± 7.2 B	**46.9 B**
5 MAP	18.8 ± 1.0 B	19.6 ± 2.6	19.5 ± 3.9 C	26.4 ± 4.0 B	19.8 ± 2.7 B	19.8 ± 2.4 BC	**20.6 D**
6 MAP	30.0 ± 1.1 B	28.8 ± 5.2	39.5 ± 11.3 BC	24.9 ± 8.4 B	44.3 ± 16.4 AB	36.6 ± 13.1 BC	**34.0 C**
12MAP	106.1± 17.9 A	55.8± 17.1	117.1± 9.4 A	118.5 ± 24.8 A	65.8 ± 13.7 A	88.7± 17.1 A	**92.0 A**
**Mean**	**42.6**	**31.3**	**51.9**	**47.1**	**38.9**	**42.0**	
**F-test**	19.19	2.96	22.00	10.51	3.65	8.19	**46.96**
***P*-value**	< 0.01	ns	< 0.01	< 0.01	< 0.05	< 0.01	**< 0.01**
**Petiole dry weight (g plant^{-1})**							
3 MAP	6.8 ± 0.9 Bd	9.2 ± 2.6 Cb-d	8.1 ± 2.4 Ccd	12.9 ± 3.9 Ca-d	15.1 ± 1.8 BCa-c	11.7 ± 3.2 a-d	**10.6 C**
4 MAP	12.5 ± 1.7 B	7.9 ± 2.7 C	16.8 ± 3.5 BC	13.8 ± 3.0 C	12.6 ± 2.7 C	15.1 ± 2.7	**13.1 C**
5 MAP	51.5 ± 11.5 A	20.4 ± 3.4 B	34.0 ± 4.6 B	41.5 ± 6.2 A	44.1 ± 13.3 AB	33.0 ± 13.0	**37.4 B***
6 MAP	54.0 ± 14.9 A	32.8 ± 4.0 A	57.7 ± 12.0 A	36.6 ± 4.2 AB	53.9 ± 17.2 A	41.8 ± 16.7	**46.1 A**
12MAP	19.4 ± 5.4 B	23.9 ± 3.3 AB	18.4 ± 1.9 BC	27.8 ± 2.5 B	4.4 ± 1.2 C	17.1 ± 2.8	**18.5 C**
**Mean**	**28.8**	**18.8**	**27.0**	**26.5**	**26.0**	**23.7**	
**F-test**	6.40	9.78	10.04	9.53	4.80	1.78	**25.91**
***P*-value**	< 0.01	< 0.01	< 0.01	< 0.01	< 0.05	ns	**< 0.01**
**Stem dry weight (g plant^{-1})**							
3 MAP	55.2 ± 5.8 C	44.6 ± 5.7 C	53.1 ± 5.2 C	61.3 ± 14.2 D	44.8 ± 9.2 C	59.5 ± 8.6 C	**53.1 D**
4 MAP	88.7 ± 7.1 C	76.2 ± 20.3 C	66.6 ± 8.5 BC	67.9 ± 12.7 D	80.0 ± 3.3 C	82.8 ± 12.0 C	**77.0 D**
5 MAP	218.8 ± 44 ACab	98.0 ± 18.4 Cd	169.5 ± 25 Ba-d	233.1 ± 39 Cab	266.5 ± 68 BCa	199.1 ± 39 BCa-c	**197.5 C**
6 MAP	453.0 ± 110.3 B	239.9 ± 26.9 B	360.1 ± 69.4 A	455.8 ± 29.4 B	547.0 ± 158.3 A	380.4 ± 67.8 AB	**406.0 A**
12MAP	728.8 ± 44.8 Aab	679.8 ± 58 Aa-c	427.4 ± 16.8 Ade	788.8 ± 24.4 Aa	347.6 ± 46.7 ABe	505.8 ± 121 Ab-e	**579.7 A**
**Mean**	**308.95**	**227.75**	**215.37**	**321.42**	**257.22**	**245.56**	
**F-test**	24.50	70.56	24.86	139.15	6.58	8.79	**72.86**
***P*-value**	< 0.01	< 0.01	< 0.01	< 0.01	< 0.01	< 0.01	**< 0.01**
**Root dry weight (g plant^{-1})**							
3 MAP	1.8 ± 0.2	2.1 ± 0.9 B	2.1 ± 0.3 B	1.0 ± 0.5 C	1.5 ± 0.6 BC	1.3 ± 0.5	**1.6 C**
4 MAP	1.5 ± 0.2 bc	0.7 ± 0.2 Bc	1.6 ± 0.3 BCbc	3.1 ± 0.7 ABab	3.6 ± 0.7 ABa	1.3 ± 0.4 bc	**2.0 BC***
5 MAP	2.2 ± 0.3 b	0.9 ± 0.3 Bb-d	2.0 ± 0.4 ABCb-d	4.4 ± 1.0 Aa	4.8 ± 0.6 ABa	2.1 ± 0.5 bc	**2.7 AB****
6 MAP	2.8 ± 0.4 b	2.4 ± 0.3 ABb	3.9 ± 0.6 Aab	3.1 ± 0.6 ABb	5.9 ± 1.1 Aa	2.8 ± 0.8 b	**3.5 A**
12MAP	2.2 ± 0.3 c	4.3 ± 1.1 Ab	0.8 ± 0.1 Cc	1.5 ± 0.1 BCc	0.8 ± 0.1 Cc	1.4 ± 0.4 c	**1.9 BC**
**Mean**	**2.15**	**2.16**	**2.11**	**2.66**	**3.36**	**1.84**	
**F-test**	2.30	4.27	7.00	4.11	7.82	1.27	**5.80**
***P*-value**	ns	< 0.05	< 0.01	< 0.05	< 0.01	ns	**< 0.01**
**Tuber dry weight (g plant^{-1})**							
3 MAP	20 ± 13 Cef	20 ± 6 Cef	22 ± 3 Bd-f	73 ± 17 Da-c	71 ± 17 Da-d	87 ± 29 Bab	**49.1 D**
4 MAP	134 ± 32 BCbc	162 ± 69 Cbc	228 ± 48 Bab	201 ± 52 Da-c	114 ± 41 Dbc	140 ± 23 Bbc	**163.5 D**
5 MAP	399 ± 120 Bb-d	322 ± 27 Cb-d	356 ± 121 Bb-d	735 ± 59 Ca	399 ± 52 Cb-d	346 ± 127 Bb-d	**426.5 C**
6 MAP	1345 ± 189 Abc	1101 ± 191 Bb-e	1543 ± 334 Aab	2064 ± 116 Aa	987 ± 125 Bb-e	919 ± 277 Ac-e	**1326.8 B****
12MAP	1377.5 ± 63.2 A	1812.5 ± 401.8 A	1534.1 ± 169.9 A	1708.1 ± 71.0 B	1474.1 ± 114.3 A	1368.8 ± 149.7 A	**1545.8 A**
**Mean**	**655.45**	**683.68**	**736.95**	**956.52**	**609.33**	**572.47**	
**F-test**	39.01	14.06	17.42	158.55	54.70	13.10	**109.01**
***P*-value**	< 0.01	< 0.01	< 0.01	< 0.01	< 0.01	< 0.01	**< 0.01**
**Total dry weight (g plant^{-1})**							
3 MAP	101 ± 17 Cde	92 ± 8 Cde	106 ± 6 Bc-e	167 ± 35 Ca-c	151 ± 17 Ca-d	175 ± 26 Cab	**132.5 D**
4 MAP	278 ± 31 Cb	283 ± 81 Cb	376 ± 63 Bab	333 ± 53 Cab	255 ± 43 Cb	289 ± 26 Cab	**302.7 D**

Parameter	Plant biomass of cassava						Mean
	RY9	RY72	KU50	CMR38-125-77	CMR35-91-63	CM523-7	
5 MAP	690 ± 123 Bbc	461 ± 25 Cc	581 ± 138 Bbc	1041 ± 100 Ba	734 ± 99 Bbc	600 ± 138 Cbc	**685.0 B**
6 MAP	1884 ± 242 Ab	1405 ± 188 Bb-d	2004 ± 386 Aab	2584 ± 101 Aa	1638 ± 303 Abc	1381 ± 323 Bb-d	**1816.6 B****
12MAP	2234.1 ± 102.4 A	2576.5 ± 420.5 A	2097.9 ± 162.1 A	2644.8 ± 88.8 A	1892.8 ± 111.4 A	1982.0 ± 38.9 A	**2238.0 A**
**Mean**	**1038.04**	**963.77**	**1033.39**	**1354.29**	**934.87**	**885.72**	
**F-test**	54.26	24.28	22.39	221.22	27.01	23.64	**150.03**
***P*-value**	< 0.01	< 0.01	< 0.01	< 0.01	< 0.01	< 0.01	**< 0.01**
**Partitioning index (PI)**							
3 MAP	0.16 ± 0.08 Ccd	0.20 ± 0.04 Cb-d	0.21 ± 0.05 Db-d	0.42 ± 0.04 Da	0.45 ± 0.08 BCa	0.44 ± 0.11 a	**0.31 D**
4 MAP	0.46 ± 0.05 Bab	0.51 ± 0.08 Bab	0.58 ± 0.04 BCab	0.56 ± 0.08 Cab	0.39 ± 0.10 Cab	0.48 ± 0.06 ab	**0.50 C**
5 MAP	0.54 ± 0.08 ABab	0.69 ± 0.05 Aab	0.56 ± 0.08 Cab	0.70 ± 0.02 ABab	0.55 ± 0.08 BCab	0.51 ± 0.13 ab	**0.59 BC**
6 MAP	0.71 ± 0.05 Aa-d	0.77 ± 0.03 Aa-c	0.76 ± 0.04 Aa-c	0.79 ± 0.02 Aab	0.63 ± 0.06 ABcd	0.63 ± 0.06 cd	**0.72 A**
12MAP	0.61 ± 0.01 ABb	0.68 ± 0.04 Aab	0.72 ± 0.03 ABab	0.64 ± 0.02 BCab	0.77 ± 0.02 Aa	0.68 ± 0.06 ab	**0.68 AB**
**Mean**	**0.50**	**0.57**	**0.57**	**0.62**	**0.56**	**0.55**	
**F-test**	11.95	20.24	17.58	11.16	4.21	1.30	32.40
***P*-value**	< 0.01	< 0.01	< 0.01	< 0.01	< 0.05	ns	< 0.01
**Leaf area index (LAI)**							
3 MAP	0.33 ± 0.11 C	1.30 ± 0.37 B	0.54 ± 0.21 B	0.60 ± 0.17 B	0.78 ± 0.10 C	0.51 ± 0.22 C	**0.68 D**
4 MAP	1.96 ± 0.23 B	1.38 ± 0.03 B	1.58 ± 0.17 B	1.41 ± 0.20 B	1.65 ± 0.28 C	1.60 ± 0.37 AB	**1.60 C**
5 MAP	4.24 ± 0.39 Aab	4.25 ± 0.12 Aab	4.56 ± 0.62 Aa	4.23 ± 0.29 Aab	4.57 ± 0.52 Ba	4.32 ± 0.61 BCa	**4.36 B***
6 MAP	4.74 ± 0.57 Aa-e	5.04 ± 0.50 Aa-d	4.64 ± 0.37 Aa-e	4.69 ± 1.06 Aa-e	6.61 ± 1.07 Aab	6.04 ± 1.71 Aa-c	**5.29 A**
**Mean**	**2.82**	**2.99**	**2.83**	**2.73**	**3.40**	**3.12**	
**F-test**	31.30	36.87	29.07	12.84	19.15	7.30	95.65
***P*-value**	< 0.01	< 0.01	< 0.01	< 0.01	< 0.01	< 0.01	< 0.01
				**Drought**			
**Leaf dry weight (g plant^{-1})**							
3 MAP	20.7 ± 4.6 BC	16.0 ± 2.4 B	30.1 ± 3.2 BC	19.1 ± 2.7 A	17.7 ± 3.7 B	11.9 ± 3.3 C	**19.3 D**
4 MAP	44.1 ± 18.2 AB	52.4 ± 9.2 AB	53.7 ± 8.1 AB	65.3 ± 23.0 AB	32.3 ± 5.3 B	38.7 ± 9.2 B	**47.8 B**
5 MAP	13.7 ± 1.8 C	14.6 ± 0.9 B	20.9 ± 5.1 C	14.8 ± 2.4 C	16.4 ± 3.2 B	19.5 ± 3.5 BC	**16.6 D**
6 MAP	24.9 ± 5.4 BC	29.3 ± 10.0 B	31.1 ± 5.3 BC	32.9 ± 7.1 BC	35.0 ± 7.5 B	30.7 ± 3.5 BC	**30.7 C**
12MAP	61.8 ± 6.1 A	88.1 ± 25.0 A	65.7 ± 15.0 A	85.8 ± 11.5 A	66.9 ± 10.2 A	72.5 ± 14.7 A	**73.4 A**
**Mean**	**33.0**	**40.1**	**40.3**	**43.6**	**33.6**	**34.7**	
**F-test**	4.57	5.79	4.87	6.54	9.67	8.20	**37.32**
***P*-value**	< 0.05	< 0.01	< 0.05	< 0.01	< 0.01	< 0.01	**< 0.01**
**Petiole dry weight (g plant^{-1})**							
3 MAP	6.9 ± 1.6 d C	7.0 ± 1.0 Bd	16.1 ± 1.1 Bab	12.0 ± 2.4 a-d	19.1 ± 3.8 BCba	9.3 ± 1.3 b-d	**11.8 C**
4 MAP	16.2 ± 6.2 BC	12.9 ± 3.5 B	13.9 ± 2.8 B	22.8 ± 9.0	7.7 ± 1.7 C	10.1 ± 3.1	**13.9 C**
5 MAP	26.7 ± 8.2 AB	13.4 ± 3.2 B	24.7 ± 5.3 B	23.0 ± 2.4	24.2 ± 6.1 B	26.6 ± 5.0	**23.1 B**
6 MAP	29.1 ± 4.8 AB	32.9 ± 7.5 A	38.8 ± 6.6 A	25.2 ± 7.4	42.5 ± 9.2 A	27.9 ± 3.1	**32.7 A**
12MAP	34.5 ± 2.4 A	12.9 ± 2.1 B	20.0 ± 3.5 B	28.2 ± 5.3	19.9 ± 2.3 BC	48.8 ± 22.6	**27.4 AB**
**Mean**	**22.7**	**15.8**	**22.7**	**22.2**	**22.7**	**24.6**	
**F-test**	4.37	5.83	5.19	1.05	5.51	2.34	**10.79**
***P*-value**	< 0.05	< 0.01	< 0.01	ns	< 0.01	ns	**< 0.01**
**Stem dry weight (g plant^{-1})**							
3 MAP	45.1 ± 3.5 C	47.1 ± 10.6 C	46.1 ± 4.0 D	71.2 ± 10.2 C	48.3 ± 6.6 D	52.5 ± 4.7 C	**51.7 D**
4 MAP	55.0 ± 9.4 C	61.3 ± 10.4 BC	92.9 ± 16.9 CD	73.1 ± 7.1 C	137.4 ± 26.5 CD	70.2 ± 4.5 C	**81.6 D**
5 MAP	156 ± 39 BCb-d	110.1 ± 13.4 BCcd	153.5 ± 24.8 Cb-d	178.0 ± 20.6 Ca-d	181.8 ± 30 Ca-d	204.0 ± 32 BCa-c	**164.0 C**
6 MAP	264.5 ± 69.8 B	223.6 ± 30.9 B	295.3 ± 56.9 B	343.5 ± 38.3 B	340.3 ± 55.4 B	383.2 ± 76.5 B	**308.4 B**
12MAP	505.9 ± 63.1 Ab-e	623 ± 122.0 Aa-d	451.2 ± 46.1 Ac-e	618.7 ± 87.1 Aa-d	648.1 ± 54.8 Aa-d	653.9 ± 115 Aa-d	**583.5 A**
**Mean**	**205.4**	**213.1**	**207.8**	**256.9**	**271.2**	**272.7**	
**F-test**	17.23	17.65	21.69	27.46	35.91	15.44	**118.37**
***P*-value**	< 0.01	< 0.01	< 0.01	< 0.01	< 0.01	< 0.01	**< 0.01**
**Root dry weight (g plant^{-1})**							
3 MAP	2.0 ± 0.1 BC	2.5 ± 0.6 BC	0.9 ± 0.3 B	2.0 ± 0.4 B	1.7 ± 0.4 B	0.9 ± 0.1	**1.7 B**
4 MAP	0.6 ± 0.1 Cc	0.5 ± 0.1 Dc	0.3 ± 0.2 Bc	0.9 ± 0.1 Bc	1.4 ± 0.4 Bbc	1.1 ± 0.4 c	**0.8 C**
5 MAP	0.6 ± 0.1 Ccd	0.7 ± 0.2 CDb-d	0.4 ± 0.2 Bd	1.2 ± 0.4 Bb-d	2.1 ± 0.7 Bbc	1.9 ± 0.8 b-d	**1.2 BC**
6 MAP	2.6 ± 0.3 ABb	3.2 ± 0.6 Bb	3.0 ± 1.0 Ab	3.3 ± 0.5 Ab	4.7 ± 1.2 Aab	2.4 ± 0.8 b	**3.2 A**
12MAP	4.1 ± 1.2 b A	7.1 ± 1.0 Aa	1.4 ± 0.2 ABc	1.5 ± 0.2 Bc	2.1 ± 0.3 Bc	2.2 ± 0.5 c	**3.0 A****
**Mean**	**2.02**	**2.81**	**1.24**	**1.82**	**2.43**	**1.71**	
**F-test**	6.61	19.03	4.22	6.30	3.29	1.17	**13.20**
***P*-value**	< 0.01	< 0.01	< 0.05	< 0.01	< 0.05	ns	**< 0.01**

Parameter	Plant biomass of cassava						Mean
	RY9	RY72	KU50	CMR38-125-77	CMR35-91-63	CM523-7	
**Tuber dry weight (g plant^{-1})**							
3 MAP	5 ± 2 Cf	19 ± 4 Def	30 ± 13 Dc-f	59 ± 17 Ba-e	96 ± 30 Ca	42 ± 5 Cb-f	**42.1 D**
4 MAP	139 ± 85 Cbc	217 ± 48 CDab	230 ± 49 CDab	292 ± 90 Ba	78 ± 24 Cc	144 ± 42 Cbc	**183.8 CD**
5 MAP	263 ± 115 BCcd	474 ± 129 Cbc	324 ± 60 Cb-d	570 ± 59 Bab	242 ± 58 Ccd	216 ± 25 Cd	**348.6 C**
6 MAP	634 ± 252 Be	1243 ± 252 Bb-d	1124 ± 103 Bb-e	1357 ± 516 Abc	766 ± 131 Bc-e	734 ± 198 Bde	**976.8 B**
12MAP	1760.5 ± 560.7 A	1776.5 ± 89.8 A	1773.4 ± 87.1 A	1813.3 ± 151.8 A	1689.4 ± 262.4 A	1582.5 ± 122.9 A	**1732.6 A****
**Mean**	**560.69**	**746.24**	**696.83**	**818.63**	**574.40**	**544.07**	
**F-test**	25.03	30.15	108.62	9.08	25.60	35.75	**128.33**
***P*-value**	< 0.01	< 0.01	< 0.01	< 0.01	< 0.01	< 0.01	**< 0.01**
**Total dry weight (g plant^{-1})**							
3 MAP	80 ± 6 Ce	92 ± 14 Dde	124 ± 16 Da-e	164 ± 25 Ba-c	183 ± 37 Ca	116 ± 8 Cb-e	**126.7 E**
4 MAP	255 ± 110 Cb	344 ± 70 CDab	391 ± 41 Cab	455 ± 125 Ba	257 ± 43 Cb	264 ± 50 Cb	**328.1 D**
5 MAP	461 ± 140 BCc	613 ± 140 Cbc	524 ± 35 Cbc	787 ± 49 ABab	466 ± 42 Cc	468 ± 25 Cc	**553.7 C**
6 MAP	955 ± 301 Bd	1532 ± 286 Bb-d	1493 ± 109 Bb-d	1762 ± 552 Abc	1189 ± 192 Bcd	1179 ± 258 Bcd	**1352.0 B**
12MAP	2366.8 ± 824.0 A	2507.9 ± 73.8 A	2311.8 ± 122.5 A	2547.4 ± 247.3 A	2426.3 ± 303.3 A	2359.9 ± 217.9 A	**2420.0 A**
**Mean**	**824.01**	**1018.14**	**969.03**	**1143.29**	**904.46**	**877.89**	
**F-test**	29.58	44.03	137.23	12.69	32.93	36.24	**181.27**
***P*-value**	< 0.01	< 0.01	< 0.01	< 0.01	< 0.01	0< 0.01	**< 0.01**
**Partitioning index (PI)**							
3 MAP	0.06 ± 0.02 Cd	0.20 ± 0.05 Cb-d	0.22 ± 0.07 Cb-d	0.33 ± 0.06 Ba-c	0.48 ± 0.07 Ba	0.36 ± 0.05 ab	**0.28 D**
4 MAP	0.41 ± 0.13 Bab	0.62 ± 0.02 Ba	0.56 ± 0.08 Bab	0.61 ± 0.05 Aa	0.27 ± 0.07 Cb	0.48 ± 0.11 ab	**0.49 C**
5 MAP	0.49 ± 0.15 ABb	0.73 ± 0.06 ABa	0.60 ± 0.07 ABab	0.72 ± 0.04 Aa	0.50 ± 0.09 ABb	0.46 ± 0.06 b	**0.58 BC**
6 MAP	0.62 ± 0.08 ABcd	0.80 ± 0.02 Aa	0.75 ± 0.04 Aa-c	0.71 ± 0.06 Aa-d	0.64 ± 0.03 ABb-d	0.57 ± 0.09 d	**0.68 AB**
12MAP	0.74 ± 0.02 Aab	0.71 ± 0.05 ABab	0.76 ± 0.01 Aab	0.71 ± 0.02 Aab	0.68 ± 0.03 Aab	0.67 ± 0.02 ab	**0.71 A**
**Mean**	**0.46**	**0.61**	**0.58**	**0.62**	**0.52**	**0.51**	
**F-test**	6.76	32.78	13.94	11.62	6.59	2.75	30.26
***P*-value**	< 0.01	< 0.01	< 0.01	< 0.01	< 0.01	ns	< 0.01
**Leaf area index (LAI)**							
3 MAP	0.81 ± 0.32 B	0.72 ± 0.23 B	0.74 ± 0.15 B	1.12 ± 0.54 B	0.44 ± 0.16 C	0.73 ± 0.17 C	**0.76 C**
4 MAP	1.25 ± 0.25 B	1.11 ± 0.12 B	1.29 ± 0.28 B	1.37 ± 0.10 B	1.15 ± 0.20 C	1.00 ± 0.18 BC	**1.19 C***
5 MAP	3.06 ± 0.28 Ab-d	2.92 ± 0.18 Acd	3.38 ± 0.31 Aa-c	2.85 ± 0.31 Acd	4.38 ± 0.41 Ba	2.40 ± 0.28 Bd	**3.16 B**
6 MAP	2.59 ± 0.64 Ae	3.62 ± 0.55 Ade	4.07 ± 0.57 Ac-e	3.02 ± 0.61 Ade	6.64 ± 1.23 Aa	4.41 ± 0.84 Ab-e	**4.06 A**
**Mean**	**1.93**	**2.09**	**2.37**	**2.09**	**3.15**	**2.14**	
**F-test**	7.04	19.35	19.41	4.94	19.12	13.58	49.48
***P*-value**	< 0.01	< 0.01	< 0.01	< 0.05	< 0.01	< 0.01	< 0.01

Each capital letter at each plant age represents the mean of six genotypes. Different capital letters indicated significant ($p < 0.05$) differences among different plant age, whereas those among genotypes are denoted with different lowercase letters. The significantly different ($p < 0.05$ and $p < 0.01$) between water regimes are donated * and **, respectively.

Table S8. Statistical analysis of photosynthetic parameters of six cassava genotypes at the plant age of 3, 4, 5, 6 and 12 months after planting (MAP). The plants were grown under different water managements.

Parameter	Plant age	Water regimes (W)			Genotypes (G)			W x G		
		df	F test	*P*-value	*df*	F test	*P*-value	*df*	F test	*P*-value
Fv/Fm	3 MAP	1	1.250	ns	5	1.220	ns	5	0.260	ns
	4 MAP	1	0.200	ns	5	1.480	ns	5	0.500	ns
	5 MAP	1	8.890	ns	5	2.490	ns	5	0.360	ns

Parameter	Plant age	Water regimes (W)			Genotypes (G)			W x G		
		df	F test	*P*-value	*df*	F test	*P*-value	*df*	F test	*P*-value
	6 MAP	1	0.250	ns	5	0.520	ns	5	0.240	ns
	12 MAP	1	0.670	ns	5	0.310	ns	5	0.390	ns
**φPSII**	3 MAP	1	6.300	ns	5	0.810	ns	5	0.400	ns
	4 MAP	1	0.000	ns	5	1.530	ns	5	1.690	ns
	5 MAP	1	0.060	ns	5	3.620	$p < 0.05$	5	1.590	ns
	6 MAP	1	1.140	ns	5	1.720	ns	5	1.630	ns
	12 MAP	1	0.920	ns	5	2.320	ns	5	0.860	ns
**ETR**	3 MAP	1	0.010	ns	5	0.580	ns	5	0.400	ns
	4 MAP	1	0.000	ns	5	0.720	ns	5	1.440	ns
	5 MAP	1	0.000	ns	5	1.160	ns	5	1.260	ns
	6 MAP	1	1.030	ns	5	2.300	ns	5	1.060	ns
	12 MAP	1	0.790	ns	5	1.620	ns	5	1.370	ns
**Pn**	3 MAP	1	0.660	ns	5	2.300	ns	5	2.330	ns
	4 MAP	1	42.970	$p < 0.01$	5	0.870	ns	5	0.980	ns
	5 MAP	1	245.590	$p < 0.01$	5	3.320	$p < 0.05$	5	1.480	ns
	6 MAP	1	1.400	ns	5	2.180	ns	5	0.660	ns
	12 MAP	1	39.670	$p < 0.01$	5	14.200	$p < 0.01$	5	5.070	$p < 0.01$
**gs**	3 MAP	1	2.000	ns	5	2.160	ns	5	0.840	ns
	4 MAP	1	52.950	$p < 0.01$	5	0.870	ns	5	1.030	ns
	5 MAP	1	14.540	$p < 0.05$	5	2.310	ns	5	0.930	ns
	6 MAP	1	1.050	ns	5	3.530	ns	5	0.300	ns
	12 MAP	1	13.430	ns	5	2.460	ns	5	0.290	ns
**Tr**	3 MAP	1	3.820	ns	5	1.970	ns	5	1.320	ns
	4 MAP	1	44.350	$p < 0.01$	5	0.330	ns	5	0.530	ns
	5 MAP	1	6.760	ns	5	2.120	ns	5	1.910	ns
	6 MAP	1	1.440	ns	5	1.890	ns	5	0.600	ns
	12 MAP	1	29.030	$p < 0.05$	5	2.610	$p < 0.05$	5	0.280	ns
**WUE**	3 MAP	1	7.010	ns	5	0.800	ns	5	1.680	ns
	4 MAP	1	16.170	$p < 0.05$	5	0.080	ns	5	0.050	ns
	5 MAP	1	0.490	ns	5	2.420	ns	5	1.670	ns
	6 MAP	1	0.220	ns	5	0.720	ns	5	1.870	ns
	12 MAP	1	1.660	ns	5	0.690	ns	5	0.900	ns

Table S9. Statistical analysis of sugar and starch of leaf, stem and of six cassava genotypes at the plant age of 3, 4, 5, 6 and 12 months after planting (MAP). The plants were grown under different water managements.

Parameter	Plant part	Plant age	Water regimes (W)			Genotypes (G)			W x G		
			df	F−test	*P*−value	*df*	F−test	*P*−value	*df*	F−test	*P*−value
Reducing sugar	Leaf	3 MAP	1	0.720	ns	5	0.380	ns	5	0.630	ns
		4 MAP	1	0.290	ns	5	2.820	$p < 0.05$	5	1.450	ns
		5 MAP	1	0.010	ns	5	2.050	ns	5	0.300	ns
		6 MAP	1	0.050	ns	5	1.950	ns	5	1.270	ns
		12 MAP	1	109.440	$p < 0.01$	5	1.840	ns	5	2.020	ns
	Stem	3 MAP	1	1.280	ns	5	3.790	$p < 0.05$	5	3.540	$p < 0.05$
		4 MAP	1	2.060	ns	5	0.250	ns	5	1.480	ns
		5 MAP	1	31.300	$p < 0.05$	5	0.620	ns	5	1.610	ns
		6 MAP	1	0.260	ns	5	0.110	ns	5	0.730	ns
		12 MAP	1	40.840	$p < 0.05$	5	6.750	$p < 0.01$	5	2.510	ns
	Tuber	3 MAP	1	0.210	ns	5	1.380	ns	5	1.720	ns
		4 MAP	1	0.370	ns	5	0.490	ns	5	0.610	ns
		5 MAP	1	10.940	ns	5	0.990	ns	5	0.550	ns
		6 MAP	1	1.970	ns	5	0.960	ns	5	1.020	ns
		12 MAP	1	0.830	ns	5	1.710	ns	5	0.360	ns
Non-reducing sugar	Leaf	3 MAP	1	0.010	ns	5	0.400	ns	5	0.210	ns
		4 MAP	1	0.030	ns	5	1.790	ns	5	0.430	ns
		5 MAP	1	3.960	ns	5	1.290	ns	5	0.930	ns
		6 MAP	1	0.420	ns	5	2.980	$p < 0.05$	5	0.660	ns
		12 MAP	1	0.630	ns	5	2.190	ns	5	0.450	ns
	Stem	3 MAP	1	47.450	$p < 0.05$	5	3.810	$p < 0.05$	5	1.900	ns
		4 MAP	1	0.120	ns	5	0.910	ns	5	0.760	ns
		5 MAP	1	4.480	ns	5	1.030	ns	5	0.690	ns
		6 MAP	1	1.340	ns	5	0.670	ns	5	0.580	ns
		12 MAP	1	0.010	ns	5	1.400	ns	5	1.480	ns
	Tuber	3 MAP	1	3.710	ns	5	1.270	ns	5	1.960	ns
		4 MAP	1	10.580	ns	5	1.080	ns	5	0.840	ns
		5 MAP	1	0.060	ns	5	0.360	ns	5	1.240	ns
		6 MAP	1	2.770	ns	5	1.910	ns	5	0.490	ns
		12 MAP	1	1.070	ns	5	17.490	$p < 0.01$	5	2.880	$p < 0.05$
Total sugar	Leaf	3 MAP	1	0.460	ns	5	2.630	ns	5	1.640	ns
		4 MAP	1	0.130	ns	5	0.550	ns	5	0.510	ns
		5 MAP	1	2.170	ns	5	2.620	ns	5	0.790	ns
		6 MAP	1	0.260	ns	5	2.850	$p < 0.05$	5	1.710	ns

Parameter	Plant part	Plant age	Water regimes (W)			Genotypes (G)			W x G		
			df	F−test	*P*−value	*df*	F−test	*P*−value	*df*	F−test	*P*−value
		12 MAP	1	1.820	ns	5	1.490	ns	5	2.950	$p < 0.05$
	**Stem**	3 MAP	1	0.970	ns	5	5.460	$p < 0.01$	5	3.320	$p < 0.05$
		4 MAP	1	0.430	ns	5	0.580	ns	5	0.550	ns
		5 MAP	1	6.380	ns	5	0.580	ns	5	1.150	ns
		6 MAP	1	1.410	ns	5	0.870	ns	5	1.540	ns
		12 MAP	1	2.670	ns	5	2.300	ns	5	0.400	ns
	**Tuber**	3 MAP	1	1.310	ns	5	1.380	ns	5	1.540	ns
		4 MAP	1	6.640	ns	5	1.960	ns	5	1.670	ns
		5 MAP	1	0.890	ns	5	0.290	ns	5	1.110	ns
		6 MAP	1	4.470	ns	5	1.110	ns	5	1.170	ns
		12 MAP	1	1.480	ns	5	5.960	$p < 0.01$	5	0.700	ns
**Starch**	**Leaf**	3 MAP	1	0.770	ns	5	4.390	$p < 0.01$	5	3.210	$p < 0.05$
		4 MAP	1	11.390	ns	5	1.030	ns	5	1.210	ns
		5 MAP	1	8.410	ns	5	1.060	ns	5	0.800	ns
		6 MAP	1	11.380	ns	5	0.520	ns	5	0.640	ns
		12 MAP	1	1.090	ns	5	4.320	$p < 0.01$	5	0.690	ns
	**Stem**	3 MAP	1	2.000	ns	5	3.630	0.017	5	2.260	ns
		4 MAP	1	0.480	ns	5	1.290	ns	5	2.710	ns
		5 MAP	1	0.270	ns	5	2.140	ns	5	1.040	ns
		6 MAP	1	10.640	ns	5	1.460	ns	5	0.610	ns
		12 MAP	1	5.560	ns	5	16.050	$p < 0.01$	5	1.320	ns
	**Tuber**	3 MAP	1	0.390	ns	5	1.180	ns	5	0.570	ns
		4 MAP	1	47.170	$p < 0.05$	5	2.080	ns	5	1.520	ns
		5 MAP	1	0.850	ns	5	1.260	ns	5	0.830	ns
		6 MAP	1	0.610	ns	5	0.820	ns	5	1.170	ns
		12 MAP	1	30.220	$p < 0.05$	5	4.540	$p < 0.01$	5	1.090	ns

Table S10. Statistical analysis of leaf, petioles, stem, root, tuber, total dry weight, harvest index (HI) and leaf area index (LAI) of six cassava genotypes at the plant age of 3, 4, 5, 6 and 12 months after planting (MAP). The plants were grown under different water managements.

Biomass	Plant age	Water regimes (W)			Genotypes (G)			W x G		
		df	F test	*P*-value	*df*	F test	*P*-value	*df*	F test	*P*-value
**Leaf DW**	3 MAP	1	0.380	ns	5	2.170	ns	5	0.690	ns
	4 MAP	1	0.010	ns	5	1.440	ns	5	1.170	ns
	5 MAP	1	8.380	ns	5	0.640	ns	5	1.050	ns
	6 MAP	1	0.900	ns	5	0.690	ns	5	0.330	ns
	12 MAP	1	2.370	ns	5	1.460	ns	5	2.140	ns
**Petiole DW**	3 MAP	1	0.630	ns	5	4.150	$p < 0.01$	5	1.300	ns
	4 MAP	1	0.090	ns	5	1.500	ns	5	1.330	ns
	5 MAP	1	10.810	$p < 0.05$	5	2.170	ns	5	0.590	ns
	6 MAP	1	5.090	ns	5	2.010	ns	5	0.600	ns
	12 MAP	1	3.990	ns	5	2.210	ns	5	2.120	ns
**Stem DW**	3 MAP	1	0.370	ns	5	1.970	ns	5	0.510	ns
	4 MAP	1	0.250	ns	5	2.430	ns	5	2.940	$p < 0.05$
	5 MAP	1	1.240	ns	5	3.610	$p < 0.05$	5	0.760	ns
	6 MAP	1	4.020	ns	5	2.380	ns	5	0.860	ns
	12 MAP	1	0.010	ns	5	3.230	$p < 0.05$	5	3.250	$p < 0.05$
**Root DW**	3 MAP	1	0.020	ns	5	1.280	ns	5	1.020	ns
	4 MAP	1	16.840	$p < 0.05$	5	8.660	$p < 0.01$	5	3.400	$p < 0.05$
	5 MAP	1	44.680	$p < 0.01$	5	7.390	$p < 0.01$	5	2.560	$p < 0.05$
	6 MAP	1	0.120	ns	5	3.650	$p < 0.05$	5	0.450	ns
	12 MAP	1	121.690	$p < 0.01$	5	16.390	$p < 0.01$	5	1.250	ns
**Tuber DW**	3 MAP	1	0.960	ns	5	5.840	$p < 0.01$	5	0.960	ns
	4 MAP	1	0.730	ns	5	4.130	$p < 0.01$	5	0.620	ns
	5 MAP	1	0.840	ns	5	6.030	$p < 0.01$	5	1.240	ns
	6 MAP	1	45.410	$p < 0.01$	5	5.020	$p < 0.01$	5	1.250	ns
	12 MAP	1	39.730	$p < 0.01$	5	0.880	ns	5	0.290	ns
**Total DW**	3 MAP	1	0.830	ns	5	4.820	$p < 0.01$	5	0.980	ns
	4 MAP	1	0.530	ns	5	2.730	$p < 0.05$	5	0.610	ns
	5 MAP	1	4.540	ns	5	4.720	$p < 0.01$	5	1.420	ns
	6 MAP	1	80.690	$p < 0.01$	5	3.740	$p < 0.01$	5	1.340	ns
	12 MAP	1	8.040	ns	5	1.660	ns	5	0.680	ns
**Harvest index (HI)**	3 MAP	1	2.65	ns	5	9.60	$p < 0.01$	5	0.41	ns
	4 MAP	1	0.01	ns	5	4.94	$p < 0.01$	5	0.78	ns
	5 MAP	1	0.01	ns	5	4.32	$p < 0.01$	5	0.22	ns
	6 MAP	1	1.52	ns	5	4.12	$p < 0.01$	5	0.49	ns

Biomass	Plant age	Water regimes (W)			Genotypes (G)			W x G		
		df	F test	*P*-value	*df*	F test	*P*-value	*df*	F test	*P*-value
	12 MAP	1	2.19	ns	5	1.96	ns	5	3.05	$p < 0.05$
**Leaf area index (LAI)**	3 MAP	1	0.16	ns	5	1.40	ns	5	2.29	ns
	4 MAP	1	31.51	$p < 0.05$	5	0.85	ns	5	0.86	ns
	5 MAP	1	16.40	$p < 0.05$	5	2.94	$p < 0.05$	5	1.45	ns
	6 MAP	1	9.08	ns	5	4.99	$p < 0.01$	5	0.67	ns
